# The ‘time’ in lifetime: age-stratification and its impact on immune-based depression treatment

**DOI:** 10.1192/j.eurpsy.2025.351

**Published:** 2025-08-26

**Authors:** E. Van Assche, C. Hohoff, B. T. Baune

**Affiliations:** 1 Department of Psychiatrie, University of Münster, Münster, Germany; 2 Department of Psychiatry, University of Melblourne; 3 Department of Psychiatry, The Florey Institute of Neuroscience and Mental Health, Melbourne, Australia

## Abstract

**Introduction:**

Depression is increasingly linked to immunological processes. Therefore, immune-based therapies, e.g., celecoxib, are being tested as augmenting treatment strategies. Many physiological processes during life are also linked to immunological changes. We tested the hypothesis that age affects treatment efficacy in a randomized controlled sample treated with the anti-inflammatory agent Celecoxib.

**Objectives:**

We test the combined role of age and an anti-inflammatory augmentation treatment for treatment response in depression. For a more in depth understanding we investigated the role of six methylation-based cell-types in these immunological processes in a second step.

**Methods:**

113 individuals with a diagnosis of major depressive disorder were included in our analyses (*M*_age_=44, 56% women, *M*_MADRS_= 27.7). All patients were treated with Vortioxetine and recruited stratified by high sensitive C-Reactive Protein (hsCRP; <= 3 vs. > 3 mg/L)> 3mg/L). Based on a randomized controlled design, augmentation with Celecoxib was administered to 55 patients. A second assessment was performed after 6 weeks of treatment (*M*_MADRS 6W_= 20.2). Cell type compositions of neutrophils, monocytes, B-cells, CD4+ and CD8+ Lymphocytes, and natural killer cells (NK), were estimated based on epigenome-wide DNA methylation markers (Illumina Infinium MethylationEPIC 850k BeadChip) using the Houseman method. Analyses were performed with linear regression models with MADRS_6W_ as outcome. Our hypothesis was tested in the full sample. The additional analyses were performed stratified by age. All models were corrected for sex, hsCRP, and depression severity at baseline.

**Results:**

Our analysis showed a statistically significant interaction between age and treatment condition on depression outcome (*p*=0.040), with significant main effects for both variables in the model (intervention: *p*=0.045, age: *p*=0.022). Sex and hsCRP were no statistically significant contributors. The intersection was identified at 45.5 years. Younger individuals treated with celecoxib showed a more pronounced reduction in MADRS (*M*_reduction_=-9.6), than older individuals treated with the same condition (*M*_reduction_=-5.5). The stratified individuals younger than 45 years, showed that neutrophils were associated with better treatment outcome (*p*=0.028), whereas for individuals older than 45 years this was the case for B-cells and NK cells (*p*=0.011, and *p*<0.001, respectively).

**Conclusions:**

Our results indicate that immunological profiles in depression and in relation to treatment may be age-dependent, which can have major consequences for treatment success with anti-inflammatory augmenting strategies. Replication in an independent sample is needed to confirm the role of age in immune-focused treatment strategies for depression.

**Disclosure of Interest:**

None Declared

